# Cross-cultural adaptation and psychometric evaluation of the Brazilian version of the Temporal Experience of Pleasure Scale (TEPS-Br)

**DOI:** 10.47626/2237-6089-2020-0131

**Published:** 2021-11-09

**Authors:** Pedro A. Rosar, Jefferson Traebert, Manuella P. Kaster, Alexandre F. Bello, André C. Haviaras, David E. Gard, Alexandre P. Diaz

**Affiliations:** 1 Universidade do Sul de Santa Catarina Palhoça SC Brazil Programa de Pós-Graduação em Ciências da Saúde, Universidade do Sul de Santa Catarina (UNISUL), Palhoça, SC, Brazil.; 2 Universidade do Sul de Santa Catarina Curso de Medicina Palhoça SC Brazil Curso de Medicina, Universidade do Sul de Santa Catarina (UNISUL), Palhoça, SC, Brazil.; 3 Universidade Federal de Santa Catarina Departamento de Bioquímica Florianópolis SC Brazil Departamento de Bioquímica, Universidade Federal de Santa Catarina (UFSC), Florianópolis, SC, Brazil.; 4 Universidade Federal do Rio Grande do Sul Hospital de Clínicas de Porto Alegre Porto Alegre RS Brazil Hospital de Clínicas de Porto Alegre, Universidade Federal do Rio Grande do Sul (UFRGS), Porto Alegre, RS, Brazil.; 5 San Francisco State University Department of Psychology San Francisco CA USA Department of Psychology, San Francisco State University, San Francisco, CA, USA.; 6 University of Texas Health Science Center at Houston Louis A. Faillace, MD, Department of Psychiatry and Behavioral Sciences Houston TX USA Louis A. Faillace, MD, Department of Psychiatry and Behavioral Sciences, University of Texas Health Science Center at Houston, Houston, TX, USA.

**Keywords:** Anhedonia, psychiatric status rating scales, validation study

## Abstract

**Introduction:**

Anhedonia is a critical symptom of major depressive disorder that is defined as the reduced ability to experience pleasure. The Temporal Experience of Pleasure Scale (TEPS) is commonly used to measure anhedonia and has exhibited satisfactory reliability.

**Objectives:**

We aim to perform cross-cultural adaptation of a Brazilian version of the TEPS and evaluate its psychometric properties.

**Method:**

The cross-cultural adaptation was performed according to previously established protocols. Cronbach’s alpha coefficient of internal consistency was used to establish the degree of interrelation and coherence of items. Also, we calculated the intraclass correlation coefficient to determine the stability of the scale after a proposed interval had elapsed and used exploratory factor analysis to evaluate the scale’s factor structure and content validity. Principal component analysis was used to determine the factors to be retained in the factor model.

**Results:**

The participants reported that the Brazilian version of the TEPS had good comprehensibility and applicability. The results revealed a statistically significant correlation between measures. The intraclass correlation coefficient calculated was significant. The Cronbach’s alpha value calculated indicated that the scale’s overall internal consistency was adequate.

**Conclusion:**

The Portuguese version of the TEPS scale proposed achieved good comprehensibility for the Brazilian population and its psychometric characteristics demonstrated good reliability and validity.

## Introduction

Anhedonia can be briefly defined as a reduced ability to experience pleasure.^1^ While being a core symptom of major depressive disorder,^2^ anhedonia is also a dimensional and transdiagnostic construct identified in a wide range of diseases such as schizophrenia, obsessive-compulsive disorder, autism spectrum disorder, and substance use disorders.^3 – 8^

Studies have shown associations between anhedonia and worse psychiatric outcomes. In a clinical trial including adolescents with treatment-resistant depression, presence of anhedonia was a predictive factor for delayed remission, regardless of other psychiatric symptoms.^9^ Anhedonia was also reported as one of the strongest predictors of poor psychosocial functioning in a study with 1570 outpatients treated for depression.^10^ In substance use disorders, anhedonia was associated with higher alcohol craving, and higher opioid abstinence, as well as social maladjustment and poor general health.^8^

Several studies have also presented an association between anhedonia and suicidal ideation. A recent meta-analysis showed that individuals with suicidal ideation had higher anhedonia scores, even in the absence of depression diagnosis.^11^ A similar association was reported by Ballard et al. in a post-hoc analysis of three clinical trials designed to evaluate the effects of ketamine.^12^ Anhedonia was associated with suicidal thoughts and one day after ketamine administration the improvement in anhedonia was associated with a reduction in suicidal ideation, even after adjustment for depressive symptoms. The authors suggested the reduction in anhedonia may be responsible, at least partially, for the antisuicidal effects of ketamine.^12^ Additionally, studies with both pharmacological treatment and non-invasive brain stimulation have reported anhedonia as a predictor of treatment response.^13 , 14^

The most common ways of measuring anhedonia are based on administration of self-report scales or questionnaires. The Temporal Experience of Pleasure Scale (TEPS) is a short, self-administered scale with satisfactory reliability, which was originally proposed by Gard and colleagues as a measure of hedonic capacity. Moreover, this scale also evaluates the existence of different subcomponents of the hedonic experience, such as the anticipatory and consummatory phases.^15^ Anticipatory pleasure is characterized by the individual’s expectation of receiving a pleasurable reward and consummatory pleasure describes the feeling of satisfaction in response to the reward. These primarily temporal differences in the subcomponents of reward have been studied in animal models and in humans since the 1970s^4^ and are associated with different underlying neurobiological mechanisms.^16^ The TEPS presents 18 items, 10 of them related to the experience of anticipatory pleasure, and the other 8 related to the ability to experience pleasure at the time of action. It also has temporal stability in test-retest assessment and validity for measurement of both anticipatory and consummatory aspects of the hedonic behavior.^15^

There is an increasing body of multinational and multicultural research, for which adapted measuring tools are needed.^17^ The TEPS has already been adapted and validated for use in several countries, including China,^18^ France,^19^ Germany,^20^ and Italy.^21^ However, there is a lack of psychometric instruments in Portuguese for measuring anhedonia in research contexts. Thus, this study aimed to perform cross-cultural adaptation of a Brazilian version of the TEPS and evaluate its psychometric properties.

## Methods

This cross-sectional study was conducted in two stages: a cross-cultural adaptation of the scale followed by evaluation of its psychometric properties.

### Cross-cultural adaptation

The cross-cultural adaptation was performed according to previously established protocols.^17 , 22 , 23^ Two independent translations into Portuguese were performed by two different researchers: one aware of the theoretical context surrounding the anhedonia theme and the other unaware of the nature of the research, both of them fluent in the original language and natives of Brazil. The researchers then merged the translations into a single document, considering their points in common and discussing possible disagreements. From this synthesis, two back-translations were performed by two independent professionals, native to the original language and culture, fluent in Brazilian Portuguese and blind to the purpose of the study.

Afterward, a committee of physicians was formed, including one with clinical experience in psychiatry and epidemiological research, three resident physicians in psychiatry, and two psychiatrists with research experience. This committee reviewed the clarity and comprehensibility of the document and ensured that the translated version described the same content as the original version. In addition, the semantic, idiomatic, experimental, and conceptual aspects of the translations were judged. At this stage, the back-translations were discussed and sent to the original author (Dr. David E. Gard) whose comments were also considered. The “pre-final” version of the TEPS was defined at this meeting, after discussion of all discrepancies by the evaluation committee.

In the second phase, 42 outpatients from the psychiatric clinics at the Polydoro Ernani de São Thiago University Hospital (HU/UFSC) were enrolled for the pre-final phase and administration of the scale. These participants were interviewed to examine their understanding of each item, as well as the answers provided. This step was conducted in order to ensure that the version retained equivalence to the circumstances. After each item, the participant was asked questions such as “Explain in your own words what you understood this question to mean,” and “Were there any words in this question you did not understand?” Additionally, the participants were also asked: “Do you have any suggestions or are there any other words we could replace for ones you didn’t understand?” All responses given during the process were noted. A final version was produced after the applicability analysis, taking the participants’ input into consideration.

### Analysis of psychometric properties

The final scale was administered to a population of 173 adult volunteers. They were all undergraduate medical students attending the second to the eleventh semester of medical school. This phase was conducted from March to April 2019 and a ratio of 10 participants per scale item was used to define the sample size.^24^ Concomitantly with the application of the final version, demographic data were collected and the HADS was administered for convergent validity testing.^25^ A subsample of approximately 20% of the participants was invited for a repeat administration of the TEPS after a seven-day interval.

Cronbach’s alpha coefficient of internal consistency was calculated and used to establish the degree of interrelation and coherence of items. The intraclass correlation coefficient was also calculated and used to determine the stability of the scale after the interval. Exploratory factor analysis was used to evaluate the factor structure and scale content after adequacy of the data had been verified with the Kaiser-Meyer-Olkin test, Bartlett’s test of sphericity, and correlation matrix analysis. After examination of the Scree plot and Kaiser criteria (eigenvalues > 1.0), principal component analysis was used to determine the number of factors to be retained in the factor model. The best solution was then subjected to orthogonal rotation (Varimax) to test the relationship between dimensions.^26^ IBM^®^ SPSS^®^ version 18.0, Microsoft^®^ Excel^®^ for Office 365 version 1904, R^®^ Core Team 2019 psych, and ISwR software packages were used to analyze and present data.

This research project was submitted through the Plataforma Brasil and approved by the Research Ethics Committee at the Universidade do Sul de Santa Catarina (protocol 3.188.771) and by the Research Ethics Committee at the Universidade Federal de Santa Catarina (protocol 36351614.2.0000.5355). All participants provided written informed consent. The authors report no conflicts of interest.

## Results

### Cross-cultural adaptation

A total of 42 outpatients from the psychiatric clinics at the HU/UFSC University Hospital were invited to participate in this phase. Two of them were excluded because of cognitive impairment, which was one of the exclusion criteria. Thus, our final sample included 40 participants. Sociodemographic variables for the participants are listed in Table 1 . Most were female (57.5%), mean age was 41±11years, and mean number of years spent in education was 11±5. On average, the patients completed the pre-final Brazilian Portuguese version of the TEPS in 12 minutes.

**Table 1:** Sociodemographic characteristics and TEPS scores of the participants included in the cross-cultural adaptation (n = 40)

Variables		N (%) / mean±SD
Gender n (%)		
	Male	17 (42.5%)
	Female	23 (57.5%)
Age (years)		40.93±11.07
Education (years)		11.43±5.02
TEPS scores		70.75±15.06

SD = standard deviation, TEPS = Temporal Experience of Pleasure Scale.

Semantics were responsible for one of the main issues observed in this phase of the study, which was also discussed with the author of the original scale. One example of this was observed in the analysis of the expression “looking forward.” In Portuguese, some translations of this expression could be interpreted as negative experiences. However, it was considered that one translation option, “aguardo ansiosamente,” generally indicates a wait for a pleasant experience in the target culture.

Thirty-five percent of the participants included in the study reported difficulty understanding item 6 of the pre-final version: “Aguardar com expectativa por uma experiência prazerosa é por si só prazeroso.” Of them, one participant suggested the removal of the expression “por si só” from the sentence, resulting in the following sentence “Aguardar por experiências prazerosas já é prazeroso.” Moreover, a total of 42.5% of the participants misinterpreted the sentence in item 13 “Não fico empolgado para coisas como ir comer em restaurantes,” from the original version: “I don’t look forward to things like eating out at restaurants.” 76.5% of the individuals who misunderstood this item suggested removing the adverb of negation “não” from the sentence, which would be problematic with regard to the inverse nature of the item. However, the negative sentence structure was maintained in the Portuguese version since it is present in this form in the original scale. The final version, generated after applicability analysis and consideration of input from the participants is presented as online-only supplementary material .

### Analysis of the psychometric characteristics

It had been planned to enroll 180 individuals to participate in this phase, a total of 10 individuals for each item on the scale. However, the population actually evaluated was 173 participants (9.6 participants per item) after seven exclusions because of incorrect or incomplete questionnaires. The sociodemographic characteristics of the participants in this phase are listed in Table 2 . The population was 73.6% female and the mean age of these participants was 22.6±4.2 years. Analysis of the adequacy of the data for validity measurements showed that the Kaiser-Meyer-Olkin criterion for sample adequacy was 0.788. Bartlett’s test of sphericity returned a significant value of p < 0.001.

**Table 2:** Sociodemographic characteristics of participants included in psychometric evaluation of the scale (n = 173)

Variables		N (%) / mean±SD
Gender n (%)		
	Male	41 (23.7)
	Female	132 (73.6)
Age (years)		22.6±4.2
Medical school semester *		
	Second	30
	Third	2
	Fourth	39
	Fifth	28
	Sixth	31
	Seventh	
	Eighth	11
	Ninth	19
	Tenth	1
	Eleventh	12

SD = standard deviation.

* In Brazil the undergraduate Medical School course has 12 periods, each of six months’ duration.

**Figure 1:**
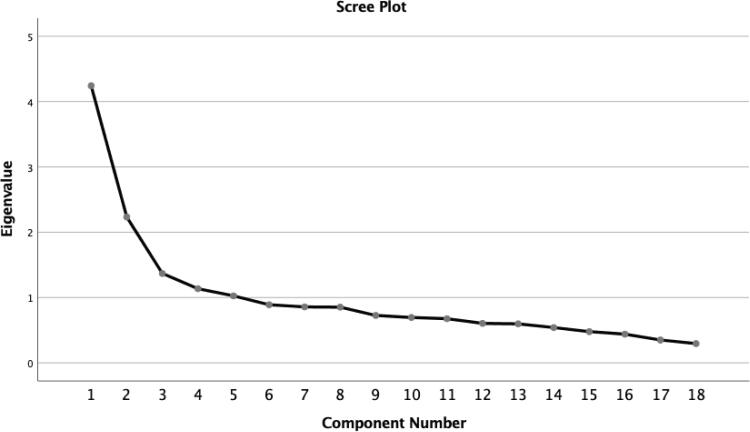
Scree plot for the factor analysis of the 18 items.

The analysis for extraction of the principal components of the Brazilian version of the TEPS initially generated five factors with eigenvalues greater than 1, although the factors were not very consistent. Two of the factors had fewer than five items with weights exceeding 0.5, and several items had weights exceeding 0.32 loading onto more than one factor.^26^ Analysis of the point of curve inflection on the Scree plot indicated that three latent factors retained information most appropriately. A three-factor model was then constructed by orthogonal rotation (Varimax), enabling reduction to three factors responsible for explaining 43.5% of total variance of the scale, with eigenvalues greater than or close to one. The eigenvalue results for the three principal components are illustrated in Figure 1 .

Table 3 shows the components of each item with their respective weights loading onto each of the three factors. The mean value of communalities was 0.435±1.26.

**Table 3:** Factor components of each item after orthogonal rotation by the Varimax method

Scale item	Factor 1	Factor 2	Factor 3
Item 1 *		0.364	0.372
Item 2	0.702		
Item 3	0.621		
Item 4 *		0.693	0.341
Item 5	0.269		
Item 6	0.298	0.492	
Item 7	0.590		
Item 8			0.595
Item 9	0.548	0.303	
Item 10			0.624
Item 11			0.730
Item 12	0.228	0.228	0.512
Item 13		-0.700	
Item 14	0.677		
Item 15		0.681	0.212
Item 16		0.684	0.282
Item 17	0.699		
Item 18 *		0.345	0.648

* Items with weights > 0.32 on more than one factor (crossloads).

### Reliability measures

**Figure 2:**
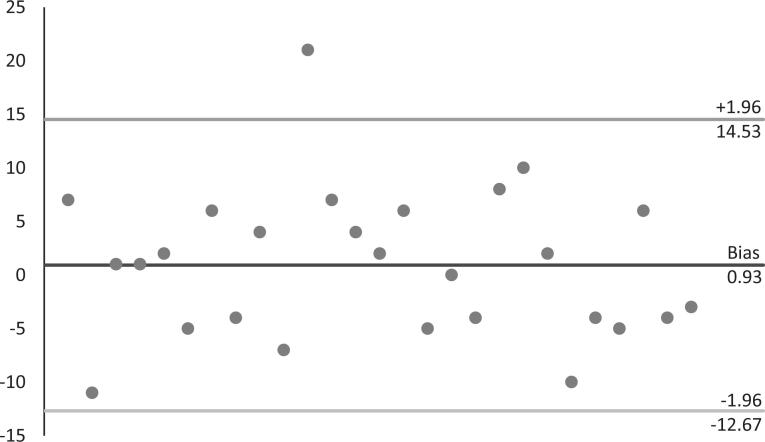
Bland-Altman plot showing deviations of the results with relation to two standard deviations.

Regarding the scale’s stability, Pearson’s coefficients were calculated for the correlation between the sum of the results of the first evaluation and the sum of the results of the second evaluation in a sample of approximately 20% of the participants retested after one week. The results revealed a statistically significant correlation between the measures (r = 0.833; p < 0.001). Calculation of the intraclass correlation coefficient returned a value of 0.815 (p < 0.001). The mean difference found in the Bland-Altman test was 0.93 ( Figure 2 – differences within a range of two standard deviations). Examination of the overall internal consistency of the scale revealed a Cronbach’s alpha of 0.745. Table 4 presents the recalculated values if each item was removed. The mean value was 0.734±0.013.

**Table 4:** Correlation between each item of the TEPS, total scores, and the Cronbach’s alpha values after exclusion of each item

Items	Correlation between each item of the TEPS and the total score *	Cronbach’s alpha value after exclusion of the item
Item 1	0.350	0.732
Item 2	0.463	0.722
Item 3	0.319	0.735
Item 4	0.405	0.730
Item 5	0.192	0.749
Item 6	0.274	0.739
Item 7	0.372	0.730
Item 8	0.361	0.731
Item 9	0.413	0.729
Item 10	0.300	0.737
Item 11	0.380	0.729
Item 12	0.447	0.723
Item 13	-0.295	0.781
Item 14	0.313	0.736
Item 15	0.437	0.725
Item 16	0.473	0.725
Item 17	0.420	0.726
Item 18	0.400	0.731

TEPS = Temporal Experience of Pleasure Scale.

* Pearson’s correlation coefficients.

The correlation analysis between the Brazilian version of the TEPS and the HADS (depression symptoms subscale) was r = -0.33.

## Discussion

The TEPS instrument was originally constructed in English language. The process of cross-cultural adaptation and psychometric analysis generated a Portuguese version of the scale for use in the Brazilian population. The Brazilian version of the TEPS has the same 18 items as the original scale. It also showed ease of application and good participant acceptance and comprehension. The proposed version was created based on the combined contributions of the expert committee and suggestions from the participants of the adaptation phase.

Although there is no defined protocol to perform a process of adaptation and validation of these scales, there is sufficient evidence suggesting that this process involves not only a simple translation, but comprehensively encompasses all the cultural diversity of the target population. We can illustrate the complexity involved in maintaining the proposed concept of the TEPS using the following item text as an example: “I appreciate the beauty of a fresh snow fall.” Freely translated into Portuguese this becomes: “Eu aprecio a beleza do cair da neve.” Since this climatic phenomenon is rare and is restricted to very few regions of Brazil, it was imperative to adopt a term that would be universal to the target population, evoking an enjoyable experience of a relationship with nature, and would maintain the same proposed construct. The phrase was therefore adapted for Brazil as: “Eu aprecio a beleza de um por do sol” [I appreciate the beauty of a sunset]. Additionally, a suggestion made by one participant in the adaptation phase was accepted by the team and the term “por si só” [in itself] was excluded, simplifying the sentence while maintaining the characteristics of item six on the scale. It can be stated that the Brazilian version of the TEPS demonstrated adequate item internal consistency, with an overall Cronbach’s alpha coefficient of 0.745 and only slight compromise to the score if each item was removed, showing good homogeneity.

It is important to note that the original scale was proposed to measure a theoretical construct that encompasses two components,^15^ one referring to experiencing pleasure in the present (consummatory pleasure) and another that refers to the prospect of experiencing pleasure in the future (anticipatory pleasure). In contrast, the Brazilian version had three main components. The first component grouped items related to pleasure experienced at the time of action, with equivalence to the original scale of consummatory pleasure. However, the items related to anticipation of pleasure were subdivided into two factors.

A similar phenomenon was observed by Chan et al. during the process of adapting the TEPS to the Chinese culture.^18^ Their adaptation also generated a subdivision of anticipatory pleasure with similar features. One component involved more subjective and imaginary processes, assessing the ability to experience pleasure in a group, and the other grouped items that describe more objective experiences. This led the authors to name the first as contextual and the second as abstract. This proposition was contested by Ho et al. two years later, who tested the four-factor model in a broad population in both England and Australia, arguing that two factors fit the construct better.^27^ Nevertheless, it is interesting to note that Li et al. reanalyzed the Chinese two-dimensional model in 2018, demonstrating temporal stability as well as invariability of internal consistency.^28^ The three constructs found in our study also illustrate the need for cross-cultural adaptations and the importance of not applying an instrument originally from other countries without following all procedures for its proper translation and validation for the new language and culture.

Thus, this final version of the Brazilian TEPS similarly expresses this concept regarding the meaning of the anticipatory subgroup. According to Ho et al.,^27^ the TEPS would be better retained with a two-factor model. However, these factors are not yet clearly defined, as shown in work by Favrod et al., who found two factors for exploratory factor analysis in their French version of the TEPS.^19^ Moreover, it is clear that just like the original scale, the Brazilian version of the TEPS is suitable for use in the Brazilian cultural context, given the clear differentiation of the theoretical constructs of experience of pleasure. It maintained the dichotomy between the same eight items regarding pleasure experienced at the time of action, as well as the same ten items concerning pleasure experienced in the future.

Regarding items that did not have considerable influence on the variance, item 12 proved to be problematic: “Eu gosto muito da sensação de um bom bocejo” [I really enjoy the feeling of a good yawn], which had the most significant effect in factor three (anticipatory) and a weak effect (below 0.3) in the conceptually adequate factor (consummatory pleasure). Interestingly, our population was composed of young adults, university students who were evaluated in the university environment. In this context such behavior (yawning) could be considered by this population as a sign of drowsiness, of laziness, or even of a lack of respect for each other. Thus, further studies with different populations are needed to better understand the role of this item. Ho et al. found that this item loaded onto both factors with weights below 0.32.^27^

Furthermore, item 18 influenced both components two and three, which is less problematic, since both components measure aspects related to anticipatory pleasure. A similar phenomenon occurred with items one and four which were considered crossloaded, i.e., they load onto more than one factor with weights greater than 0.3.^26^ Additional testing is expected to better clarify the value of these items for the scale set. Statistical techniques such as Item Response Theory (IRT) can verify how crossloaded items add information to the instrument. Posteriorly, confirmatory factor analysis could test the correlations between the three factors defined in the cross-cultural adaptation.

Despite the fact that 42.5% of the population studied in the process of cross-cultural adaptation had difficulty perceiving the negative nature of item 13, it was maintained according to the original scale. It is observed that this is the only negative sentence in the whole scale. Despite the rejection expressed in the adaptation phase, this same item exhibited a strong ability to express the variance of component two in the component analysis, as well as an adequate relationship with the other items. Obviously, since it is a negation, it is represented by a negative sign in the factor analysis. For this reason, this item was kept in this Brazilian version of TEPS with the same negative verbal structure and the inverse measurement when compared to the other items.

The reliability analysis of the Brazilian version of the TEPS showed a significant intraclass correlation coefficient (p < 0.001) with a value of 0.815. In addition, the difference in the test-retest results showed pertinent dispersion in the Bland Altman plot, with the results encompassed within a range of two standard deviations and only one outlier.

Analysis of the correlation between TEPS scores and HADS scores was non-significant. However, there was a weak correlation (r = -0.33) with the depression symptoms subscale (even items on the general scale), which shows a closer approximation between the anhedonia construct and the depression construct, when measured by the HADS. Nevertheless, it is considered that this result reveals two different constructs, and a stronger correlation would hardly be expected. Similar correlations were found in other studies that have compared these same characteristics and corroborate this observation.^19 , 20^ Only studies using scales that specifically measured the anhedonia construct found stronger correlations.^15 , 27^ It is suggested that this type of analysis may be performed in the future for the Brazilian population as soon as such tools have had their adaptations and validity tested for use in this population.

One limitation of this study is the lack of better concurrent and convergent validity testing. Another is the fact that the population studied in the second phase is not representative of the general population. However, as a strength, we can highlight the cross-cultural adaptation with a clinical sample of patients followed-up in public psychiatric outpatient care. The Brazilian version of the TEPS proposed achieved good comprehensibility and applicability. In addition, its psychometric characteristics demonstrate reliability and validity. Thus, the Brazilian version of the Temporal Scale of Pleasure Experience (TEPS-Br) is proposed as an instrument for evaluation of hedonic capacity in the Brazilian population.

## Supplementary material


