# Supplementary material for: Cross-cultural adaptation and psychometric evaluation of the Brazilian version of the Temporal Experience of Pleasure Scale (TEPS-Br)
Source: Trends Psychiatry Psychother. 2021 Nov 9;44:e20200131. doi: 10.47626/2237-6089-2020-0131 (PMC9911165; doi:10.47626/2237-6089-2020-0131)
Supplement: Supplementary file 1 [file 2238-0019-trends-44-e20200131-suppl01.pdf]

http://dx.doi.org/10.47626/2237-6089-2020-0131
Cross-cultural adaptation and psychometric evaluation of the Brazilian version of the Temporal Experience of Pleasure Scale (TEPS-Br) - Rosar et al.

Escala Temporal de Experiências Prazerosas

INSTRUÇÕES: Por favor, leia cuidadosamente cada afirmação e decida o quão verdadeiro são para você essas afirmações de forma geral. Por favor, responda *todos os itens*. Caso você ***nunca*** tenha tido a experiência descrita, pense sobre a experiência mais parecida que você já tenha tido e escolha sua resposta. *Não* deixe questões em branco. Escolha somente *uma* resposta para cada afirmação. Não se preocupe em manter a consistência entre as suas respostas. Escolha uma das 6 (seis) opções seguintes e **CIRCULE** sua resposta no espaço à direita do item.

**1**	**2**	**3**	**4**	**5**	**6**
Muito falso para mim	Moderadamente falso para mim	Um pouco falso para mim	Um pouco verdadeiro para mim	Moderadamente verdadeiro para mim	Muito verdadeiro para mim

1.	Quando eu fico sabendo de um novo filme estreado pelo meu ator favorito, eu mal posso esperar para assisti-lo.	1 2 3 4 5 6
2.	Eu gosto de inspirar profundamente o ar fresco quando eu caminho ao ar livre.	1 2 3 4 5 6
3.	O cheiro de grama recém cortada é agradável para mim.	1 2 3 4 5 6
4.	Eu aguardo com expectativa coisas em minha vida.	1 2 3 4 5 6
5.	Eu adoro quando as pessoas mexem no meu cabelo.	1 2 3 4 5 6
6.	Aguardar por experiências prazerosas já é prazeroso.	1 2 3 4 5 6
7.	É muito prazeroso para mim tomar um copo de café ou chá quente em uma manhã fria.	1 2 3 4 5 6
8.	Quando eu penso em algo saboroso, como um biscoito de chocolate, eu tenho que comer um.	1 2 3 4 5 6
9.	Eu aprecio a beleza de um pôr do sol.	1 2 3 4 5 6
10.	Eu fico tão empolgado na noite anterior a um feriado que eu mal consigo dormir.	1 2 3 4 5 6
11.	Quando eu estou a caminho de um parque de diversões, mal posso esperar para ir na montanha-russa.	1 2 3 4 5 6
12.	Eu gosto muito da sensação de um bom bocejo.	1 2 3 4 5 6
13.	Não fico empolgado para coisas como ir comer em restaurantes.	1 2 3 4 5 6
14.	Eu adoro o som da chuva na janela quando estou deitado na minha cama aconchegante.	1 2 3 4 5 6
15.	Quando eu penso em comer minha comida favorita eu quase consigo sentir seu gosto de tão bom que é.	1 2 3 4 5 6
16.	Quando eu peço alguma coisa de um cardápio, eu imagino o quão saborosa ela será.	1 2 3 4 5 6
17.	O som da lenha estalando na fogueira é muito relaxante.	1 2 3 4 5 6
18.	Quando algo empolgante está para acontecer na minha vida, eu realmente aguardo ansiosamente por isso.	1 2 3 4 5 6

http://dx.doi.org/10.47626/2237-6089-2020-0131

Escala Temporal de Experiências Prazerosas - Pontuação

INSTRUÇÕES: Por favor, leia cuidadosamente cada afirmação e decida o quão verdadeiro são para você essas afirmações de forma geral. Por favor, responda *todos os itens*. Caso você ***nunca*** tenha tido a experiência descrita, pense sobre a experiência mais parecida que você já tenha tido e escolha sua resposta. *Não* deixe questões em branco. Escolha somente *uma* resposta para cada afirmação. Não se preocupe em manter a consistência entre as suas respostas. Escolha uma das 6 (seis) opções seguintes e **CIRCULE** sua resposta no espaço à direita do item.

**1**	**2**	**3**	**4**	**5**	**6**
Muito falso para mim	Moderadamente falso para mim	Um pouco falso para mim	Um pouco verdadeiro para mim	Moderadamente verdadeiro para mim	Muito verdadeiro para mim

1.	Quando eu fico sabendo de um novo filme estreado pelo meu ator favorito, eu mal posso esperar para assisti-lo. ***(Antecipatório)***	1 2 3 4 5 6	10.	Eu fico tão empolgado na noite anterior a um feriado que eu mal consigo dormir. ***(Antecipatório)***	1 2 3 4 5 6
2.	Eu gosto de inspirar profundamente o ar fresco quando eu caminho ao ar livre. ***(Consumatório)***	1 2 3 4 5 6	11.	Quando eu estou a caminho de um parque de diversões, mal posso esperar para ir na montanha-russa. ***(Antecipatório)***	1 2 3 4 5 6
3.	O cheiro de grama recém cortada é agradável para mim. ***(Consumatório)***	1 2 3 4 5 6	12.	Eu gosto muito da sensação de um bom bocejo. ***(Consumatório)***	1 2 3 4 5 6
4.	Eu aguardo com expectativa coisas em minha vida. ***(Antecipatório)***	1 2 3 4 5 6	13.	Não fico empolgado para coisas como ir comer em restaurantes. ***(Antecipatório – codificação inversa)***	1 2 3 4 5 6
5.	Eu adoro quando as pessoas mexem no meu cabelo. ***(Consumatório)***	1 2 3 4 5 6	14.	Eu adoro o som da chuva na janela quando estou deitado na minha cama aconchegante. ***(Consumatório)***	1 2 3 4 5 6
6.	Aguardar por experiências prazerosas já é prazeroso. ***(Antecipatório)***	1 2 3 4 5 6	15.	Quando eu penso em comer minha comida favorita eu quase consigo sentir seu gosto de tão bom que é. ***(Antecipatório)***	1 2 3 4 5 6
7.	É muito prazeroso para mim tomar um copo de café ou chá quente em uma manhã fria. ***(Consumatório)***	1 2 3 4 5 6	16.	Quando eu peço alguma coisa de um cardápio, eu imagino o quão saborosa ela será. ***(Antecipatório)***	1 2 3 4 5 6
8.	Quando eu penso em algo saboroso, como um biscoito de chocolate, eu tenho que comer um. ***(Antecipatório)***	1 2 3 4 5 6	17.	O som da lenha estalando na fogueira é muito relaxante. ***(Consumatório)***	1 2 3 4 5 6
9.	Eu aprecio a beleza de um pôr do sol. ***(Consumatório)***	1 2 3 4 5 6	18.	Quando algo empolgante está para acontecer na minha vida, eu realmente aguardo ansiosamente por isso. ***(Antecipatório)***	1 2 3 4 5 6
